# Aberrant Sialylation in Cancer: Therapeutic Opportunities

**DOI:** 10.3390/cancers14174248

**Published:** 2022-08-31

**Authors:** Jennifer Munkley

**Affiliations:** Centre for Cancer, Biosciences Institute, Newcastle University, Newcastle upon Tyne NE1 3BZ, UK; jennifer.munkley@ncl.ac.uk

**Keywords:** cancer, glycosylation, glycans, sialic acid, aberrant sialylation, hypersialylation, therapeutics

## Abstract

**Simple Summary:**

All cells are covered in a thick layer of sugar molecules known as glycans. Changes to this sugar coat are common in cancer, and in particular cancer cells often display high levels of a glycan known as sialic acid. Sialic acid glycans play important roles in cancer biology and can help tumours grow, spread to other sites, and evade the immune system. Strategies to target sialic acid are being actively investigated and hold huge potential for cancer research. Here, I outline why sialic acid is so important in cancer, discuss recent advances in this field, and highlight opportunities for the development of new sialic acid targeting therapies.

**Abstract:**

The surface of every eukaryotic cell is coated in a thick layer of glycans that acts as a key interface with the extracellular environment. Cancer cells have a different ‘glycan coat’ to healthy cells and aberrant glycosylation is a universal feature of cancer cells linked to all of the cancer hallmarks. This means glycans hold huge potential for the development of new diagnostic and therapeutic strategies. One key change in tumour glycosylation is increased sialylation, both on *N*-glycans and *O*-glycans, which leads to a dense forest of sialylated structures covering the cell surface. This hypersialylation has far-reaching consequences for cancer cells, and sialylated glycans are fundamental in tumour growth, metastasis, immune evasion and drug resistance. The development of strategies to inhibit aberrant sialylation in cancer represents an important opportunity to develop new therapeutics. Here, I summarise recent advances to target aberrant sialylation in cancer, including the development of sialyltransferase inhibitors and strategies to inhibit Siglecs and Selectins, and discuss opportunities for the future.

## 1. Introduction

Glycosylation is the most common, complex, and dynamic post-translational modification of both membrane-bound and secreted proteins [1]. Glycans are fundamental to many biological processes and play a key role in protein folding, stability, trafficking, and activity, and act as regulators of signalling pathways, cell differentiation, immune recognition, and host–pathogen interactions [2,3,4]. Glycans consist of two main classes: *O*-glycans, initiated in the Golgi apparatus by the initial attachment of GalNAc moieties to serine or threonine residues to form the Tn antigen, and *N*-glycans, which are initiated in the ER via the addition of an oligosaccharide chain to asparagine residues [5,6]. In addition, intracellular proteins can be modified with O-GlcNAc [7]. Glycan chains may be branched or elongated and the cellular glycome is composed of glycans covalently linked to lipids (glycolipids and glycosphingolipids) or proteins (glycoproteins and proteoglycans). The synthesis of glycans is non-templated, meaning that glycan sequences are not directly coded by the genome [8]. Instead, glycans are produced at the tissue level and can respond dynamically to environmental stimuli and signalling molecules via the coordinated activity of biosynthetic enzymes, the trafficking of these enzymes to the endoplasmic reticulum (ER) and Golgi apparatus, and the availability of sugar donors [3]. Glycans can be conjugated to proteins and lipids, or they can be secreted without conjugation to other macromolecules. In human cells, glycans are primarily constructed from ten monosaccharides: glucose (Glc), galactose (Gal), N-acetylgalactosamine (GalNAc), N-acetylglucosamine (GlcNAc), fucose (Fuc), sialic acid (Neu5Ac), mannose (Man), xylose (Xyl), glucuronic acid (GlcA), and iduronic acid (IdoA). These monosaccharides are assembled into glycans by biosynthetic enzymes in the Golgi apparatus and the ER, and additional complexity can arise from further modifications by sulfation, phosphorylation, methylation, and acetylation. In addition to glycosylation being an intracellular event, recent studies have demonstrated that glycans can undergo further modification by extracellular enzymes, further revealing the complexity of the dynamic glycome [9,10,11].

Aberrant glycosylation in cancer was first described more than fifty years ago [12]. Since then, changes to glycans have been identified in every type of cancer [13], and altered glycosylation has been linked to all of the cancer hallmarks [3,14,15]. Many of the first cancer-specific antibodies detect oncofoetal antigens present on embryonic and cancer cells but not in adult healthy tissue [16], and numerous FDA-approved tumour markers, including CEA, CA125, and PSA, are glycan antigens or glycoproteins [17,18,19]. Common changes to the tumour glycome include aberrant sialylation, fucosylation, truncated *O*-glycans and alterations to *O*- and *N*-glycan branching. A dense layer of tumour-associated glycans coats the cell surface of cancer cells and is a driving force behind tumour growth, metastasis and immune evasion [15,20,21]. Aberrant glycosylation can interfere with cell adhesion molecules such as cadherins and integrins and alter the function of receptor tyrosine kinases (RTKs). Tumour-associated glycans can also bind to lectins, including galectins, sialic acid-binding immunoglobulin-type lectins (Siglecs) and Selectins. Glycans have functional roles in regulating cell proliferation, cell signalling, cell adhesion, extracellular matrix interactions and proximal and distal communication [3]. These biological processes play a critical role in cancer biology, and it has become evident that tumour glycosylation can have a major impact on cancer progression, tumour immunity, and clinical outcome.

Sialic acid is a key monosaccharide building block of mammalian cell-surface glycans and in humans, the most common sialic acid is N-acetylneuraminic acid (Neu5Ac). Sialic acid residues are present at the tip of glycans, positioning them at the forefront of crucial biological processes [22]. One common feature of cancer cells is increased cell-surface sialylation [22,23]. The ‘sialome’ is a subclass of the glycome [24], and has been described as a dense forest coating the cell surface in a complex array of sialylated structures that has far-reaching consequences for cancer [25]. In this review, I discuss the mechanisms behind how cancer cells become hypersialylated, how increased sialylation is advantageous to cancer cells and tumours, and highlight emerging strategies to target aberrant sialylation to develop new cancer therapeutics.

## 2. Aberrant Sialylation in Cancer

Aberrant sialylation is a prominent feature across many cancer types and has been recognised as a cancer hallmark [26,27]. The first indications that sialylation is important in tumourigenesis came from studies that discovered increased sialylation and sialyltransferase activity in malignant cells [28,29,30], and showed that the pre-treatment of cancer cells with neuraminidase can reduce engraftment and inhibit tumour growth in vivo [31,32,33]. Early studies also showed that the ability of tumour cells to metastasise correlates with total sialic acid levels [34], and it was proposed that increased sialylation in tumour cells can act as a mask to evade recognition by the immune system [29]. Sialoglycans are known to regulate glycoprotein and glycolipid structure, stability, function and trafficking, and a growing body of evidence now demonstrates how hypersialylation is advantageous to tumours. Increased sialylation of cancer cells can promote tumour growth, metastasis, immune evasion and drug resistance [23], meaning strategies to block aberrant sialylation on tumours will be highly beneficial.

Cancer patients have long been reported to express ‘Hanganutziu–Deicher’ antibodies that recognise gangliosides carrying the non-human sialic acid Neu5Gc (N-glycolylneuraminic acid), which is also detected in human tumours [15]. Neu5Gc is a major form of sialic acid in mammals but cannot be biosynthetically produced in humans due to the loss of CMP-sialic acid hydroxylase (CMAH) [35,36,37]. Neu5Gc differs from human sialic acid, Neu5Ac, by addition of a single oxygen atom and can be present in humans due to the incorporation of diet-derived Neu5Gc into human glycans [15,38]. Humans show variable levels of anti-Neu5Gc antibodies, which has been linked to tumourigenesis. Specifically, in a humanised mouse model lacking the CMAH gene, anti-Neu5Gc antibodies have been linked to an increased rate of liver cancer [35]. The primary dietary source of Neu5Gc is red meat, and it has been speculated that this may help explain the increased cancer risk associated with red meat consumption [15].

### 2.1. Sialylated Glycans

**Figure 1:**
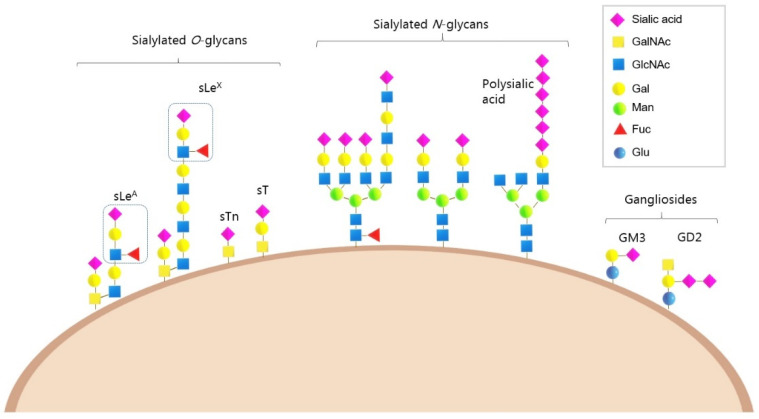
Hypersialylation is a common feature of cancer cells. Tumour cells have increased levels of sialylated glycans on their cell surface, which include sialyl Lewis^A^ (SLe^A^), sialyl-Lewis^X^ (SLe^X^), sialyl-Tn (sTn), Sialyl-T (sT), polysialic acid (polySia), GM3 and GD2 antigens.

The cell surface of cancer cells is covered in a dense layer of sialylated glycans, which can include sialyl-Tn (sTn), sialyl-T (sT) and sialyl-Lewis antigens, polysialic acid, and gangliosides [39] (Figure 1 and Table 1). These tumour-associated antigens are often exploited as markers for the detection and monitoring of cancer [15,40]. The sTn antigen is a truncated O-glycan containing a sialic acid α-2,6 linked to GalNAc and is a well-characterised cancer-associated glycan that is upregulated in virtually all epithelial cancers and associated with a poor patient outcomes [40]. sTn has been investigated widely as a circulating biomarker for several cancers, and a vaccine against sTn has been tested in clinical trials [41]. The sialyl-T (sT) antigen (Neu5Acα2-3Galβ1-3GalNAc-) is upregulated in several tumour types, including breast, ovarian, brain and renal cancers, and is associated with reduced survival times in patients [42,43,44,45,46,47].

**Figure 2:**
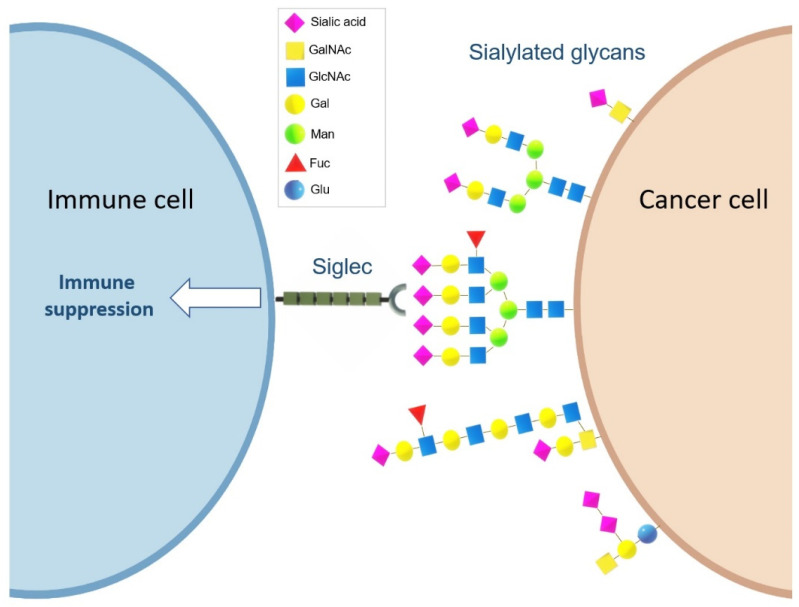
Siglec–sialoglycan interactions can modulate immune cell function and promote an immunosuppressive tumour microenvironment (TME).

Other important sialylated glycans include the sialyl Lewis antigens, sLe^A^ and sLe^X^, which are found at high levels in many solid tumours and adenocarcinomas. Both sLe^A^ and sLe^X^ are ligands for Selectins, a family of lectins that play a role in immune-cell trafficking [48]. Cancer cells displaying sLe^A^ and SLe^X^ are recognised as leucocytes, which enables them to leave the bloodstream and metastasise to other sites [49]. The cancer antigen CA 19-9 contains sLe^A^ and is routinely used to monitor treatment response in pancreatic cancer [50], and sLe^X^ is associated with a higher risk of metastasis [51,52]. Increased levels of sialylation on cancer cells also leads to upregulation of sialylated ligands that are recognised by lectin receptors known as Siglecs (Sialic acid-binding immunoglobulin-type lectins) on immune cells, and Siglec–sialoglycan interactions can modulate immune cell function and promote an immunosuppressive tumour microenvironment (TME) [53] (Figure 2).

Cancer cells also display increased expression of the α2,8-linked polymer known as polysialic acid (polySia). Upregulation of polysialic acid has been detected in several cancer types and is associated with high-grade tumours [3,22,54,55]. Polysialic acid is often present on NCAM1 (neural cell adhesion molecule 1) [54,56], and expression correlates with metastatic disease and poor clinical prognosis [57,58]. Gangliosides (sialic-acid-containing glycosphingolipids) such as the complex ganglioside GD2 can also be upregulated in cancer and are implicated in tumour development [59,60]. Strategies to therapeutically target GD2 are currently in development, including GD2-CAR T cell therapy [61] and the monoclonal antibody dinutuximab [62]. The ganglioside GM3 is also implicated in cancer and is being investigated as a target for immunotherapy [63,64]. In addition to an overall upregulation of sialylated glycans in tumour cells, there can also be changes in sialic acid modifications [15]. A growing number of studies associate sialic acid O-acetylation with cancer, and this has been linked to metastasis and tumour immunity [65,66,67].

**Table 1:** Summary of sialylation changes in cancer.

Sialylation Change	Link to Cancer	References
sialyl Tn (sTn)	sTn is upregulated in numerous epithelial cancers and associated with poor patient outcomes. sTn has been investigated as a circulating biomarker for several cancers, and the Theratope vaccine against sTn has been tested in clinical trials.	[40,41]
Sialyl-T (sT)	The sT antigen is upregulated in several tumour types, including breast, ovarian, brain and renal cancers, and is associated with reduced survival times in patients.	[42,43,44,45,46,47].
Selectin ligands	The sialyl Lewis antigens (sLe^A^ and SLe^X^) are found at high levels in many cancer types and linked to metastasis. sLe^A^ and SLe^X^ are ligands for Selectins and enable cancer cells to leave the bloodstream and colonise other organs.	[48,49,51,52]
Siglec ligands	Increased levels of sialylation on cancer cells leads to upregulation of sialylated ligands that are recognised by Siglec receptors on immune cells. Siglec–sialoglycan interactions can modulate immune cell function and promote an immunosuppressive tumour microenvironment (TME).	[53]
Polysialic acid (polySia)	Polysialic acid is often upregulated in high-grade tumours, and expression correlates with metastatic disease and poor clinical prognosis.	[3,22,54,55,57,58]
Gangliosides	The gangliosides GD2 and GM3 can be upregulated in cancer and are being actively investigated as therapeutic targets.	[59,60,61,62,63,64]

### 2.2. Sialyltransferase Enzymes

Twenty different sialyltransferase enzymes have been identified and classified into four groups: ST3GAL, ST6GAL, ST6GALNAC and ST8SIA [68,69]. These enzymes add sialic acid (Neu5Ac) to galactose (ST3GAL and ST6GAL), N-acetylgalactosamine (GalNAc) (ST6GALNAC), or sialic acid (ST8SIA) in α2-3, α2-6, or α2-8 glycosidic linkages, respectively [70]. The altered expression of sialyltransferase enzymes plays a critical role in tumour biology, and sialyltransferase enzymes have been linked to malignant disease [22]. Sialic acid can be removed from glycoconjugates by sialidase enzymes (also known as neuraminidases) [71]. There are four sialidase enzymes (NEU1-4) [72], and individual sialidases have also been associated with certain cancer types [73]. The upregulation of sialyltransferases has been linked to Ras and c-Myc signalling, as well as gene amplification, DNA methylation, hypoxia and androgen steroid hormones [74,75,76,77].

The most well-described sialyltransferase is human ST6GAL1, which adds sialic acids in an α2-6 linkage to galactose residues of Galβ1-4GlcNAc-R on *N*-glycans [75]. A second enzyme, ST6GAL2, can also add α2-6 linked sialic acid to *N*-glycans, but this enzyme is mainly expressed in the brain [78]. ST6GAL1 is overexpressed in numerous cancer types, and there is extensive literature linking ST6GAL1 to tumour grade, metastasis and poor patient prognosis [75,79,80,81,82]. ST6GAL1 mediated α2,6-linked sialylation of receptors, including the β1 integrin [83,84,85,86,87], the receptor tyrosine kinases EGFR, MET and HER2 [88,89,90] and the Fas and TNFR1 death receptors [91,92], can promote invasion and resistance to apoptosis. ST6GAL1 is also implicated in the epithelial to mesenchymal transition (EMT) [88,93] and can promote a cancer-stem-cell phenotype [80,94]. In addition, ST6GAL1 can modulate T-cell responses in the tumour microenvironment and play a role in cancer cell immune evasion [95,96].

Canonically, ST6GAL1 is localised intracellularly within the trans-Golgi network, and it is within this context that the role of ST6GAL1 in cancer biology has been interpreted. However, catalytically active ST6GAL1 is also present in extracellular spaces and systemic circulation, and extracellular ST6GAL1 is a potent modifier of processes including inflammatory cell production, haematopoiesis, B-cell differentiation and the sialylation of IgG [10,97,98,99]. Early work suggested that circulating ST6GAL1 is mainly released by the liver [100], but recent studies suggest that cancer cells also have the capacity to increase extracellular ST6GAL1 levels [11,101]. ST6GAL1 detected in patient blood has been identified as a novel biomarker for lenvatinib-susceptible FGF19-driven hepatocellular carcinoma, which could aid in optimal drug selection [101]. Excitingly, a recent study revealed that breast cancer cells release ST6GAL1 in exosome-like vesicles, and this extracellular enzyme can remodel the cell surface and secrete glycans to promote breast cancer cell growth and invasiveness [11]. This finding is consistent with previous findings that exosomes are enriched with both ST6GAL1 and α2,6-sialylated glycoproteins [102,103], and raises the intriguing possibility of targeting extracellular ST6GAL1 therapeutically.

ST3GAL1 acts predominantly on core-1 *O*-glycans and catalyses the transfer of sialic acid to a galactose residue in α2-3 linkage to generate the sialyl-T antigen from the T antigen Galβ1-3GalNAc. ST3GAL1 is overexpressed in malignant tissues, including breast [42] and ovarian cancer [43]. In breast cancer, ST3GAL1 has been shown to promote tumorigenesis [104] and is associated with poor clinical outcomes and an inflammatory phenotype [105,106]. ST3GAL1 has also been linked to immune evasion through the sialylation of CD55 [107]. In pancreatic cancer, ST3GAL1 enhances metastatic potential [108] and promotes the synthesis of ligands for Siglec-7 and Siglec-9 on tumour cells to drive tumour-associated macrophage differentiation [109]. Other members of the ST3 family, namely ST3GAL4 and ST3GAL6, have been linked to cancer [22,110,111,112,113,114]. ST3GAL4 and ST3GAL6 are both involved in the synthesis of sLe^A^ and sLe^X^ [48,115]. Cell-surface glycans containing sLe^A^ and sLe^X^ act as binding ligands for Selectins and play key roles in facilitating metastasis. ST3GAL4 upregulation promotes c-Met activation and an invasive phenotype in gastric carcinoma cells [114], and in multiple myeloma ST3GAL6 promotes homing and engraftment to the bone marrow niche and is associated with inferior overall survival in patients [111]. 

The ST6GALNAC family catalyses the glycosidic linkage of sialic acids to GalNAc (N-galactosamine) residues found on *O*-glycosylated proteins or glycolipids in an α2-6 linkage. ST6GALNAC1 adds sialic acid to *O*-linked GalNAc residues to promote the formation of the tumour-associated sialyl-Tn (sTn) antigen [116]. sTn is overexpressed in many cancer types [40,117] and is associated with poor clinical outcomes [118,119,120,121]. Upregulation of ST6GALNAC1 can promote tumour growth and metastasis [122,123,124] and is linked to cancer cell stemness [121,123]. ST6GALNAC1 can be induced by cytokines [125], and studies show that binding of sTn to Siglec-15 on macrophages can suppress T-cell responses, leading to immune evasion in the tumour microenvironment [126,127,128]. High expression of ST6GALNAC2, which synthesises the sialyl-6-T antigen and to a lesser extent sialylates the Tn antigen [116,129], has been linked to poor prognosis in colorectal cancer [130] and metastasis in thyroid cancer [131], but has been identified as a metastasis suppressor and correlated with increased patient survival times in breast cancer [132].

ST8SIA enzymes transfer a sialic acid residue to another sialic acid in α2-8 linkages, contributing to the synthesis of oligosialic and polysialic acid chains [133,134,135]. Of particular interest, 2,8-disialic structures have been shown to be ligands for Siglec-7 and Siglec-9 and may act as glycoimmune checkpoints in cancer [100,101]. ST8SIA2 and ST8SIA4 are polysialyltransferases producing polysialylated cell adhesion molecules, which are re-expressed during cancer progression [134,136,137]. ST8SIA2 correlates with tumour progression in non-small-cell lung cancer [138] and has been linked to tumour invasion and metastasis [139,140]. ST8SIA4 is overexpressed in breast and renal cell carcinoma tissues [141,142] and is linked to chemoresistance in acute myeloid leukaemia [143]. In contrast, in follicular thyroid carcinoma, ST8SIA4 is downregulated and has been shown to suppress tumour growth [144]. ST8SIA3 generates oligo-sialylated structures [133] and has been identified as a therapeutic target for glioblastomas [145]. Other ST8SIA family members, including ST8SIA1, ST8SIA and ST8SIA6, have also been linked to a malignant potential. ST8SIA1 and ST8SIA5 produce di- and tri-sialylated structures, respectively, but exclusively on gangliosides. ST8SIA1 (also known as GD3 synthase) catalyses the ganglioside GD3 [15] and has been associated with tumour growth and progression [146,147]. Decreased expression of ST8SIA5 correlates with reduced survival in patients with colorectal cancer [148] and has been linked to gene regulation by FOXO3, which may facilitate inflammation-mediated colon cancer growth [148]. ST8SIA6 transfers sialic acid onto NeuAcα2,3 (6)Gal disaccharide acceptor substrates including glycolipids and O-linked glycoproteins [149] to generate α2,8-linked disialic acids. ST8SIA6 is upregulated in many cancer types and is associated with poor prognosis [150]. Studies show that ST8SIA6 can promote tumour growth in mice by inhibiting immune responses in tumours, characterised by macrophage polarisation toward M2 and upregulation of the immune modulator arginase [150].

### 2.3. Sialidase Enzymes

Sialidases cleave sialic acids from glycoconjugates and are key enzymes controlling the sialic acid content of cells. Sialylation levels can be modified synergistically by both sialyltransferase and sialidase enzymes, and while the role of sialyltransferase enzymes in malignancy is well-explored, far fewer studies have investigated the role of sialidases in cancer. There are four mammalian sialidases, NEU1-4, and each enzyme has a distinct cellular location. NEU1 is predominantly located in lysosomes, NEU2 in the cytosol, NEU3 in the plasma membrane, and NEU4 is located in mitochondria [151]. The four human sialidases also differ in their substrate specificities and appear to have differing roles in malignancy [151]. 

Published data investigating the role of NEU1 in cancer are somewhat contradictory. In colon cancer, NEU1 has been linked to the suppression of metastasis through de-sialylation of integrin beta4 [152], and in bladder cancer, NEU1 is downregulated and can suppress in vivo tumour formation by inhibiting Akt signalling [153]. Downregulation of NEU1 can also inhibit the cell proliferation and invasion capabilities of ovarian cancer cells [154]. However, in contrast, studies have also demonstrated that NEU1 can promote pancreatic cancer metastasis [155]. NEU3 is upregulated in numerous cancer types [156,157,158,159] and contributes to tumorigenesis, most likely by modifying transmembrane signalling [160], and downregulation of NEU4 correlates with increased invasion in colon cancers [161]. These studies show that while increased sialylation of tumours is often attributed to the upregulation of sialyltransferase enzymes, sialidase enzymes are also important modulators of sialylation in cancer cells and can contribute to tumour hypersialylation.

## 3. The Functional Role of Sialylation in Cancer

### 3.1. Metastasis

Metastasis is the spread of cancer cells from the primary tumour to surrounding tissues and other organs and is the main cause of death in cancer patients [162]. Metastasis consists of several steps to enable cancer cells to leave the primary tumour mass, to intravasate and survive in the circulation, to extravasate and seed in secondary sites, and to initiate the growth of metastatic lesions. Although recent advances have shed light on the metastatic cascade [163], there is still more to uncover, in particular in relation to the role of glycosylation in metastasis. Hypersialylation is closely linked to a pro-metastatic phenotype, and sialylated glycans are critical to several processes involved in metastasis [13,25,32].

Altered adhesion between cancer cells and the extracellular matrix (ECM) and other cells in the tumour is a key mechanism that allows cells to dissociate from the primary tumour, leading to potential metastasis at secondary sites. Numerous studies have revealed a link between hypersialylation and the altered adhesion of cancer cells [25]. For example, the sialylation of integrins can modulate the adhesion, migration, and signalling of metastatic cells [85,86,87,164]. In breast cancer cells, α2,6 hypersialylation of integrin β1 decreases cell adhesion [165], and in colon cancer cells, enhanced sialylation of β1 promotes adhesion to collagen I and increases cancer cell migration [84], and decreased sialylation of integrin β4 can suppress cell migration, adhesion and invasion [152].

EMT is the process by which immobile epithelial cells transition into motile mesenchymal cells [166]. EMT involves the disruption of cell–cell adhesion and polarity, remodelling of the cytoskeleton, and changes to cell–matrix adhesion. Sialyltransferases play an integral role in EMT to promote cancer cell invasiveness and metastatic activity [167]. In breast cancer, ST6GAL1 can promote TGFβ-induced EMT as well as maintenance of a mesenchymal state [93], and in pancreatic cancer, ST6GAL1-mediated sialylation can upregulate mesenchymal markers and enhance cell invasion [168]. The ST3GAL1 enzyme has also been shown to promote cell migration, invasion, and TGF-β1-induced EMT in ovarian cancer [43,47]. In contrast, other studies show that sialylation can be downregulated during EMT, but then increased again once cells are in a mesenchymal state [169].

To metastasise, cancer cells circulating in the bloodstream or lymphatic system must ‘tether’ to cells at a secondary site by interacting with extracellular molecules, followed by ‘rolling’ of the cancer cell against endothelial cells, resulting in firm adhesion and facilitating extravasation and colonisation. The adhesion of tumour cells to endothelial cells occurs through interactions with a family of cell-adhesion molecules known as Selectins [48,115]. Selectins are classified as P-, E-, and L-selectin and are expressed on platelets, endothelial cells and leukocytes, respectively, and their ligands, sLe^A^ and sLe^X^, are found on cell-surface antigens such as CD24, CD44 and the P-selectin glycoprotein ligand (PSGL1) [3,170]. The Selectins and their ligands play a key role in cancer metastasis [115,171,172,173] and have also been linked to therapy resistance [174,175]. E-selectin ligands have been shown to promote homing to bone marrow and may play a role in the metastasis of cancer cells to bone [176]. E-selectin is also important in breast cancer, where E-selectin facilitates entry into the bone marrow niche [177], and the binding of cancer cells to E-selectin induces EMT and WNT signalling and promotes breast cancer bone metastasis [178].

### 3.2. Cancer Cell Survival

The ability of cancer cells to evade programmed cell death by apoptosis is a hallmark of cancer [179]. Glycans play an important role in many of the processes leading to apoptosis, and altered glycosylation of cell death receptors can enable cancer cells to resist cell death [180]. Hypersialylation of receptors, such as Fas and tumour necrosis factor receptor 1 (TNFR1) death receptor, can protect against apoptosis and contribute to increased cancer cell survival [91,92]. Sialylated glycans can also inhibit interactions between Galectin-3 (Gal-3) and its binding partners (which include integrins, collagen, mucins, and fibronectin) [181]. Together, these findings highlight the role of sialylated glycans in promoting cancer cell survival and raise the possibility of targeting aberrant sialylation as a therapeutic strategy to hinder the ability of cancer cells to evade apoptosis.

### 3.3. Immune Evasion

To grow and successfully metastasise, cancer cells must avoid detection and destruction by the immune system [182]. One way cancer cells achieve this is by mimicking the cell-surface glycosylation of healthy cells to employ a self signal and avoid immune attack [183]. Glycan structures on the cell surface are among the first assemblies that interact with immune cells, and the specific glycan signatures found on tumour cells, known as the tumour glyco-code, can alter how the immune system perceives cancer cells and can induce immune suppression [184]. The early evidence that tumour sialic acid promotes immune evasion came from the discovery that de-sialylated fibrosarcoma cells show decreased proliferation in immunocompetent mice but not in irradiated mice [185]. A family of lectin receptors known as Siglecs (Sialic acid-binding immunoglobulin-type lectins) have emerged as key mediators of this effect [186] and hypersialylation is now emerging as a potential new immune checkpoint [53] (Figure 2). Siglecs are primarily expressed on immune cells, such as T cells, NK cells and monocytes, and have an immunoreceptor tyrosine-based inhibitory motif [187]. Siglecs transmit inhibitory signals and are comparable with the immune checkpoint inhibitor programmed death protein 1 (PD-1) [187,188]. A recent explosion of data implicating Siglecs in cancer has made this an active area of research [188], and studies have shown that tumour cells can exploit Siglec-sialoglycan interactions to modulate immune cell function and promote an immunosuppressive tumour microenvironment (TME) [53].

Siglec-7 and -9 expression on tumour-associated macrophages (TAMs) can promote cancer progression by driving macrophage polarisation towards the M2 phenotype to establish an immunosuppressive tumour microenvironment [109,189,190]. Siglec-10 is also expressed by tumour-associated macrophages and can interact with tumour-expressed CD24 to promote immune suppression [191]. Similarly, interactions between tumour sialoglycans and Siglec-7 or Siglec-9 expressed on natural-killer (NK) cells can inhibit tumour cell death [192,193]. In mouse models of lung cancer, neutrophils that express Siglec-F (the mouse homologue to Siglecs 5 and 8) can remodel the tumour immune microenvironment and drive the growth of tumours [194]. Studies suggest that T cells can express and be negatively regulated by Siglec-5, Siglec-7, Siglec-9 and Siglec-10 [195,196], and Siglec-15 has been shown to increase tumour growth rates and suppresses antigen-specific T-cell responses [126]. Siglec-15 has also been identified as an immune suppressor. Recent findings show that Siglec-15 is upregulated in the tumour microenvironment, and its expression is mutually exclusive to PDL1 across numerous cancer types [126].

Although it was initially hypothesised that immune cells expressing Siglecs are inhibited upon binding to sialylated ligands on target cells, it has now been discovered that Siglec-15 is present on both tumour-infiltrating myeloid cells and tumour cells [126]. There are differences in Siglecs between mouse and humans [197], and moving forward, the development of models expressing human Siglecs on murine immune cells or mouse models engrafted with human immune cells [198,199,200] will increase our understanding of the role Siglecs play in the tumour microenvironment [22]. In addition to Siglecs, numerous other mechanisms have been proposed for how hypersialylation modulates the host immune response to cancer cells, including the skewing of T cell responses [96,201] and sialic-acid-mediated self-recognition by complement factor H [202].

### 3.4. Therapy Resistance

Increased sialylation of tumours can contribute to chemotherapy and radiotherapy resistance in several types of cancer, believed to be potentially due to the physical barrier of extra sialic acid on the cell surface potentially absorbing ionising radiation, modifying key receptors, and blocking the uptake of drug molecules into the cell. Altered sialylation of tumour cells has been linked to cisplatin and paclitaxel resistance in ovarian cancer [43,203,204], docetaxel sensitivity in hepatocarcinoma [205], imatinib resistance in chronic myeloid leukaemia [206], multidrug resistance in human acute myeloid leukaemia [143], chemotherapy resistance in gastric cancer [207], resistance to tyrosine kinase inhibition in lung cancer [208], and bortezomib sensitivity of myeloma cells [49]. A correlation between hypersialylation and radiotherapy resistance have also been reported, particularly in colorectal cancer [209,210,211,212]. A recent study also reported that sialylation of the oncogenic receptor Erb2 can mask the epitope of an anti-cancer antibody (trastuzumab) to promote resistance to treatment [213]. These studies raise the potential for targeting aberrant sialylation alongside existing therapies to boost treatment response and suggest that sialylated glycans can likely also be exploited to predict sensitivity and resistance to treatment strategies. As the mechanisms underlying sialic-acid-mediated drug resistance are poorly understood, further investigations in this area will be crucial to develop new therapeutic strategies to disarm drug resistance. 

## 4. Therapeutic Strategies to Inhibit Aberrant Sialylation

### 4.1. Sialyltransferase Inhibition

There are several potential strategies to block the incorporation of sialic acid onto cell-surface glycans. These include inhibition of the CMP-sialic acid transporter and inhibition of sialyltransferase enzymes. Targeting CMP-sialic acid via a specific inhibitor decreases cell-surface sialic acid and can inhibit the metastasis of colorectal cancer [214], and knockdown of the CMP-sialic acid transporter impeded the growth of melanoma tumours and suggested that hypersialylation impedes T-cell-mediated anti-tumour responses while promoting tumour-associated regulatory T cells [215]. 

Inhibition of sialyltransferase enzymes is also being pursued as a strategy to block cell-surface sialylation. The cell-permeable peracetylated 3F_ax_-Neu5Ac (P-3FAX-Neu5Ac) is a global metabolic inhibitor of sialylation [216,217]. Intracellularly, the fluorinated prodrug P-3Fax-Neu5Ac is converted into the active inhibitor CMP-3F_ax_-Neu5Ac to inhibit all sialyltransferases and reduce global sialylation. 3FAX-Neu5Ac fails to be used as a substrate by biosynthetic enzymes, and thus diminishes sialic acid content by ~80–90%. However, when P-3FAX-Neu5Ac was tested in a murine model, the global inhibition of sialylation produces liver and kidney dysfunction [218]. To overcome the deleterious effect on liver and kidney function, Bull et al. have performed targeted delivery of P-3FAX-Neu5Ac using nanoparticles to prevent metastasis in a mouse lung cancer model [219], and have also utilised intra-tumoural injection of 3Fax-Neu5Ac to suppress tumour growth in multiple cancer models by promoting T-cell-mediated immunity [201]. Despite the localised site of injection, renal toxicity was still noted at higher doses, thus highlighting the need for better-tolerated versions of 3FAX-Neu5Ac for use in vivo. Further derivatives of 3F_ax_-Neu5Ac have been developed and tested as cancer therapeutics, including C-5-modified 3-fluoro sialic acid sialyltransferase inhibitors (where the natural N-acetamide group is replaced with a carbamate functionality) [220]. These novel inhibitors are more efficiently metabolised towards their CMP analogues, reach higher effective concentrations within the cell, and induce prolonged inhibition of both α2,3 and α2,6-linked sialylation [220]. Hence, C-5 carbamate sialyltransferase inhibitors hold promise to inhibit sialylation in cancer, and future studies should explore the use of these new inhibitors in vivo.

Several natural products with the potential to inhibit sialyltransferases are available, including ginsenosides, which can inhibit both α-2,3- and α-2,6-sialylation [221], soyasaponin I, which inhibits ST3GAL1 [222,223], flavonoids that can inhibit ST6GAL1 [224], and lithocholic acid, which is active against ST3GAL1 [225,226]. Further derivatives of lithocholic acid have been developed, including the novel sialyltransferase inhibitor Lith-O-Asp, which inhibits ST3GAL1, ST3GAL3 and ST6GAL1 and can suppress metastasis [226]. Sialyltransferase inhibitors have also been identified through high-throughput screening, where lead compounds include pyrazole, which shows high selectivity towards ST3GAL3 [227]. In addition, cyclopentanoid-type compounds have also been developed and have shown promise as sialyltransferase inhibitors [228].

Due to the potential off-target effects on the liver and kidney exhibited by the pan-inhibitor 3FAX-Neu5Ac, it has been suggested that for sialyltransferase inhibitors to proceed to clinical trials, they will need to be specific to individual enzymes [25]. However, as sialyltransferase enzymes are often membrane-bound proteins, this has led to difficulties in successfully crystallising the enzymes, and only a handful of human sialyltransferase structures exist [229]. These include ST6GAL1 [230], ST6GalNAC2 [231], and ST8SIA3 [232] and have enabled the development of a new series of carbamate-linked sialyltransferase inhibitors [233]. Of particular interest, arbamate-linked uridyl-based inhibitors of human ST6GAL1 have been developed and provide a promising new class of sialyltransferase inhibitors to be further explored [233]. To date, ST3GAL1 and ST6GAL1 have been the most commonly investigated and targeted sialyltransferase enzymes in cancer. Moving forward, wider studies including the entire panel of 20 human sialyltransferases hold exciting potential to develop novel inhibitors.

### 4.2. Selectin Inhibitors

Anti-selectin antibodies, antibodies that target selectin ligands, and other platforms to block Selectin–ligand interactions are being investigated as a means of blocking cancer metastasis. Anti-Selectin antibodies in the pipeline as agents to block Selectin–ligand interactions include crizanlizumab, which blocks P-selectin [234], and specific humanized blocking antibodies for P-selectin and PSGL-1 [235]. The E-selectin inhibitor Uproleselan (GMI-1271), developed by GlycoMimetics, has shown promise for treating acute myeloid leukaemia (AML) in combination with chemotherapy (NCT02306291) [175,236,237], with phase III trial results expected in 2023 (NCT03616470), and may also re-sensitise multiple myeloma to therapy [235]. In addition, other pharmacological approaches to disrupt Selectin–ligand interactions are also being developed [238,239,240,241,242].

### 4.3. Antibody–Sialidase Conjugates

Increased sialylation helps cancer cells evade immune destruction, and targeting aberrant sialylation offers the ability to reprogram immune responses to tumours. Sialidase treatment of a variety of cancer cells has been shown to remove Siglec ligands and enhance the clearance of cancer cells injected into mice, thus highlighting an exciting opportunity to sensitise cancer cells to immunosurveillance [192,243]. Antibody–sialidase conjugates can enable the targeted destruction of self-associated sialylated glycans to enhance anti-tumour immunity [244]. Novel routes to target Siglec–sialic acid interactions include using a sialidase conjugated to a HER2 antibody (trastuzumab) to de-sialylate cancer cells [193,245]. Here, cancer cell de-sialylation can remove Siglec ligands and enhance NK cell killing of breast cancer cells. Improved antibody–enzyme conjugates have now been developed that utilise a human sialidase with improved biocompatibility and stability in vivo [246]. In syngeneic breast cancer models, the removal of sialylated glycans enhanced immune cell infiltration and activation and prolonged survival times in mice with trastuzumab-resistant breast cancer. These reagents are currently being further developed by Palleon Pharmaceuticals [188], who are evaluating an HER2–sialidase conjugate, as both a single agent and in combination with pembrolizumab (anti-PD1) in previously treated non-small-cell lung cancer, colorectal cancer, melanoma, pancreatic cancer, and ovarian cancer (NCT05259696). This exciting Phase I/II clinical trial will evaluate destroying sialylated glycan-mediated immune checkpoints in combination with traditional immune checkpoint blockade as a novel approach to overcome immune resistance in cancer. Antibody–sialidase conjugates could be applicable to other tumour types in addition to breast cancer. It is exciting to speculate that human sialidases could be conjugated to other antibodies, including, for example, a prostate-specific membrane antigen (PSMA) antibody, to selectively de-sialylate prostate cancer cells and induce an anti-tumour immune response. In addition, whereas sialidases can remove Siglec ligands, other enzymes can likely also be used to modify the cancer glycocalyx.

### 4.4. Anti-Siglec Antibodies

Monoclonal antibodies that target glycan-binding proteins have shown promise as therapeutic agents. Antibodies avoid some of the challenges of directly targeting glycans and benefit from favourable pharmacokinetics and manufacture. Anti-Siglec antibodies can potentially block Siglec–ligand interactions and modulate the function of immune cells. Several companies are pursuing strategies to create Siglec-blocking antibodies. Anti-Siglec-9 antibodies can prevent Siglec-9 inhibitions on TAMs [247] and are currently in preclinical development for cancer immunotherapy [248]. Anti-Siglec 7 antibodies have been shown to be effective at blocking Siglec–ligand interactions to promote NK-mediated killing [192], and an anti-Siglec-15 blocking antibody (NC318) is currently being tested in clinical trials (NCT03665285) [249,250].

### 4.5. Vaccines

The development of vaccines as human glycan-targeted therapeutics is an active area of research. MUC1 decorated with sTn can predict survival in ovarian cancer [251], which led to the development of the Theratope sTn-KLH vaccine [252,253]. Initial clinical trials showed that Theratope can promote the generation of antibodies against sTn [254], and the presence of anti-sTn antibodies correlates with increased patient survival [255]. However, unfortunately, a phase III clinical trial (NCT00003638) for metastatic breast cancer found no benefit for patients receiving the Theratope vaccine [256]. It is important to note that patient eligibility for the trial was not determined by tumour sTn expression, which could explain the failure of this trial. Other vaccines have since been developed, including a unimolecular pentavalent vaccine (containing glycan portions of Globo-H, GM2, sTn, TF and the Tn antigen) that increased antibody titres to these antigens in a phase I clinical trial (NCT01248273) [257]. A KLH conjugate vaccine has also been produced for sLe^A^ and tested in metastatic breast cancer patients (NCT00470574) [258].

## 5. Conclusions

**Figure 3:**
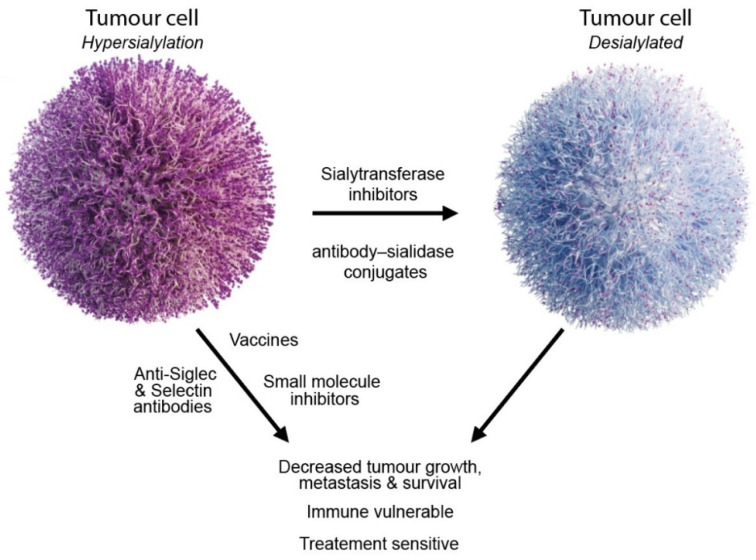
Strategies to target aberrant sialylation in cancer.

Hypersialylation is a common feature of tumours that has far-reaching consequences. Aberrant sialylation plays a key role in tumour progression by enabling evasion of cell death and immunosurveillance and by promoting metastasis. Sialylated glycans can also contribute to chemotherapy and radiotherapy resistance in several cancer types. Targeting abnormal sialylation represents an exciting strategy to develop new glycan-targeting therapeutics, and the groundwork has been laid for an explosion of therapeutic opportunities in this area. Among the most promising therapeutic agents in the pipeline are sialyltransferase inhibitors, antibodies and inhibitors targeting Siglecs and Selectins, antibody–sialidase conjugates, and vaccines (Figure 3 and Table 2). The sialome acts as an essential interface between cells and the surrounding microenvironment; however, much remains to be uncovered regarding the function of sialylated glycans in disease. New approaches to improve our ability to detect altered sialylation will catalyse the development of new glycan-targeted therapeutics. As the focus of cancer therapy moves towards precision medicine, the tumour glycome will provide clinically actionable information towards patient-tailored treatments. There is much to be gained from targeting aberrant sialylation in cancer but still so much to explore.

## Figures and Tables

**Table 2:** Overview of therapeutic strategies to target aberrant sialylation in cancer.

Approach	Progress	References
Sialyltransferase inhibitors	Intra-tumoural injection of 3Fax-Neu5Ac suppresses tumour growth in multiple cancer models by promoting T-cell mediated immunity. Targeted delivery of P-3FAX-Neu5Ac using nanoparticles can prevent metastasis in a mouse lung cancer model. However, 3Fax-Neu5Ac has been shown to produce liver and kidney dysfunction in mice. C-5 carbamate sialyltransferase inhibitors that reach higher concentrations within the cell and induce prolonged inhibition of sialylation have been developed but are yet to be tested in vivo.	[168,218,219,220]
Selectin inhibitors	Blocking antibodies for P-Selectin have been developed. The E-selectin inhibitor Uproleselan (GMI-1271) has shown promise for treating acute myeloid leukaemia (AML) in combination with chemotherapy, and may also re-sensitise multiple myeloma to therapy.	[234,235] NCT02306291 [175,235,236,237] NCT03616470
Antibody–sialidase conjugates	A sialidase conjugated to a HER2 antibody (tratuzumab) can de-sialylate cancer cells, remove Siglec ligands and prolong survival times in mice. A HER2-sialidase conjugate is currently in Phase I/II clinical trials in combination with traditional immune checkpoint blockade for patients with non-small cell lung cancer, colorectal cancer, melanoma, pancreatic cancer, and ovarian cancer.	[188,193,245,246] NCT05259696
Anti-Siglec antibodies	Anti-Siglec 9 antibodies are in preclinical development. Anti-Siglec 7 antibodies have been shown to promote NK mediated killing. The anti-Siglec-15 blocking antibody (NC318) is being tested in clinical trials.	[192,247,248,249,250] NCT03665285
Vaccines	The Theratope sTn-KLH vaccine can promote the generation of anti-sTn antibodies, but a phase III trial showed no benefit for metastatic breast cancer patients. A unimolecular pentavalent vaccine containing vaccine portions of Globo-H, GM2, sTn, TF and the Tn antigen has been tested in patients with ovarian, fallopian tube or peritoneal cancer, and produced increased antibody titres to these antigens in a phase I clinical trial. A KLH conjugate vaccine has also been produced for sLe^A^ and tested in metastatic breast cancer patients.	NCT00003638 [256] NCT01248273 NCT00470574 [257,258]
